# Dr. Charles Davis Hurt

**Published:** 1907-02

**Authors:** E. C. Davis, R. R. Kime


### OBITUARIES.

### DR. CHARLES DAVIS HURT.

Dr. Charles Davis Hurt died in the sixty-third year of his life, having accomplished more in this period than is usually allotted to man. The mature life of physicians either mollified or made harsh by the daily visitations to the beds of suffering and the complaints of the afflicted. Dr. Hurt's nature was softened, his demeanor always cheery and bright and his religious faith a benediction to those in pain and doubt. He had lived long enough to have his experience mature his judgment, and yet he was cut down before the grim ravages of age had begun to impair his professional usefulness. As a man his nature was intensive, always busy, either with his profession or his church. He was strongly religious in his tendencies and through his efforts much of the present Wesley Memorial Hospital owes its existence.

As a soldier he served through the civil war in the cavalry service largely, with credit to himself and the cause for which he fought. At the close of the war he laid aside his personal animosities and endeavored to advance the interest of his country by close application to his duties as a farmer. He began the study of medicine in 1869 under Dr. B. F. Johnson, of Notasulga, Ala. A year later he attended his first course of lectures at the Atlanta Medical College. The following year he graduated in medicine at the Medical Department of The University of Georgia, at Augusta. After graduation he practiced medicine one year at Notasulga, Ala., removing from there to Hurtsboro, Ala., where he practiced until 1884, when he moved to Columbus, practicing there for eight years, enjoying a lucrative practice and holding the confidence and esteem of a large clientele. While in Columbus he was President of the Board of Health, a member of the school board, chairman of the Board of Stewards of the St. Luke Methodist Church, etc.

In 1892, desiring a still larger field for his efforts, he came to Atlanta. He was soon made surgeon of the Street Railway System, in which position he served up to the time of his death. He was Vice-President of the Atlanta Society of Medicine and served as Censor for the Georgia Medical Association, visiting physician to the Grady Hospital and to the Wesleyan Memorial Hospital, and Professor of Therapeutics in the Atlanta School of Medicine. In all his positions he preserved a modesty worthy of a true physician, and in his loss this Society feels that they have suffered a true loss, the community deprived of a valued citizen, and in the home and family a void has been made which can never be filled.

We, therefore, request that this brief sketch be spread on the minutes of the Society, published in the Atlanta Journal-Record of Medicine and a copy sent the bereaved family.

Committee,
E. C. DAVIS,
R. R. KIME,

